# Alcohol consumption and lifetime change in cognitive ability: a gene × environment interaction study

**DOI:** 10.1007/s11357-014-9638-z

**Published:** 2014-03-21

**Authors:** Stuart J. Ritchie, Timothy C. Bates, Janie Corley, Geraldine McNeill, Gail Davies, David C. Liewald, John M. Starr, Ian J. Deary

**Affiliations:** 1 Department of Psychology, The University of Edinburgh, 7 George Square, Edinburgh, EH8 9JZ UK; 2 Centre for Cognitive Ageing and Cognitive Epidemiology, The University of Edinburgh, Edinburgh, UK; 3 Institute of Applied Health Sciences, University of Aberdeen, Aberdeen, UK; 4 Centre for Molecular Medicine, The University of Edinburgh, Edinburgh, UK; 5 Medical Research Council (MRC) Institute of Genetics and Molecular Medicine, Edinburgh, UK; 6 Alzheimer Scotland Dementia Research Centre, The University of Edinburgh, Edinburgh, UK

**Keywords:** Alcohol, Cognitive ageing, Longitudinal modelling, Genetics, Alcohol dehydrogenase, Mendelian randomization

## Abstract

Studies of the effect of alcohol consumption on cognitive ability are often confounded. One approach to avoid confounding is the Mendelian randomization design. Here, we used such a design to test the hypothesis that a genetic score for alcohol processing capacity moderates the association between alcohol consumption and lifetime change in cognitive ability. Members of the Lothian Birth Cohort 1936 completed the same test of intelligence at age 11 and 70 years. They were assessed for recent alcohol consumption in later life and genotyped for a set of four single-nucleotide polymorphisms in three alcohol dehydrogenase genes. These variants were unrelated to late-life cognition or to socioeconomic status. We found a significant gene × alcohol consumption interaction on lifetime cognitive change (*p* = 0.007). Individuals with higher genetic ability to process alcohol showed relative improvements in cognitive ability with more consumption, whereas those with low processing capacity showed a negative relationship between cognitive change and alcohol consumption with more consumption. The effect of alcohol consumption on cognitive change may thus depend on genetic differences in the ability to metabolize alcohol.

## Introduction

Findings concerning the effects of alcohol on cognitive outcomes in ageing are equivocal. Several recent review articles have concluded that consumption of alcohol at moderate levels is linked to protective effects on cognition in later life (Anstey, Mack, and Cherbuin 2009; Kim et al. 2012; Neafsey and Collins 2011), and specific biochemical mechanisms—such as the possible anti-inflammatory properties of alcohol—have been suggested to underlie this effect (Collins et al. 2009). However, some studies have found contradictory results, linking even moderate alcohol consumption to greater risk of later-life cognitive decline (see above reviews and Panza et al. 2008). The inconsistent results may be due to correlations between alcohol consumption and cognition being subject to problems of confounding and reverse causation. For example, Corley et al. (2011) found that significant positive associations between alcohol consumption and cognition in old age were almost entirely accounted for by cognitive ability measured in childhood and by socioeconomic status (SES).

Here, we further investigate the data used by Corley et al. (2011) by testing the hypothesis that the direction of the effect of alcohol on cognition depends on an individual’s genetically influenced alcohol processing capacity. We also extend some previous work that uses Mendelian randomization (MR; Davey Smith 2010; Ebrahim and Davey Smith 2008) to test the effects of alcohol consumption on cognitive ability. MR is a technique that uses, as instrumental variables, genetic variants that are not associated with the outcome variable, but instead alter the action of a putative risk factor. If the genetic variants are distributed at random with regard to confounders such as SES, an interaction of genotype with the exposure variable of interest (in the present context, alcohol) on the outcome (in the present context, cognitive ability) indicates causal effects of the exposure variable. In one such study, Au Yeung et al. (2012) examined the moderating effect of the well-studied ‘alcohol flush’ single-nucleotide polymorphism (SNP; number rs671 on the gene *ADH*) on alcohol’s effect on cognitive function in a large sample of Chinese men. *ADH*, along with related genes, encodes alcohol dehydrogenase enzymes that catalyze the breakdown of alcohol (ethanol) into acetaldehyde (Dickson et al. 2006; Birley et al. 2009). They found that individuals who received more biological exposure to alcohol due to their lower genetic propensity to metabolize it were not different in their cognitive ability levels to those with normal metabolic ability. The authors concluded that the ostensible beneficial effect of alcohol on cognition was in fact due to confounding by other factors such as SES.

However, use of other genetic variants has produced different results. Lewis et al. (2012) used a set of four SNPs in three genes (*ADH1A*, *ADH1B*, and *ADH7*) to examine the effect of maternal consumption of alcohol on offspring cognition. The total number of rare alleles on this SNP set interacted significantly with alcohol consumption: Children with more rare alleles tended to have lower cognitive ability at age 8, but only if their mothers were moderate drinkers during pregnancy. In the present study, in a longitudinal sample followed up after approximately 60 years, we test whether the same set of genetic differences in alcohol metabolism studied by Lewis et al. (2012) interact with personal alcohol consumption to affect change in an individual's own cognition from childhood to old age. Our examination of the trajectory of cognitive ageing from childhood to old age is an advantage, since few studies have examined the effects of alcohol on cognitive change before old age (see Richards, Hardy, and Wadsworth 2005, and Zanjani, Downer, Kruger, Willis, and Schaie 2013, for exceptions).

**Fig. 1:**
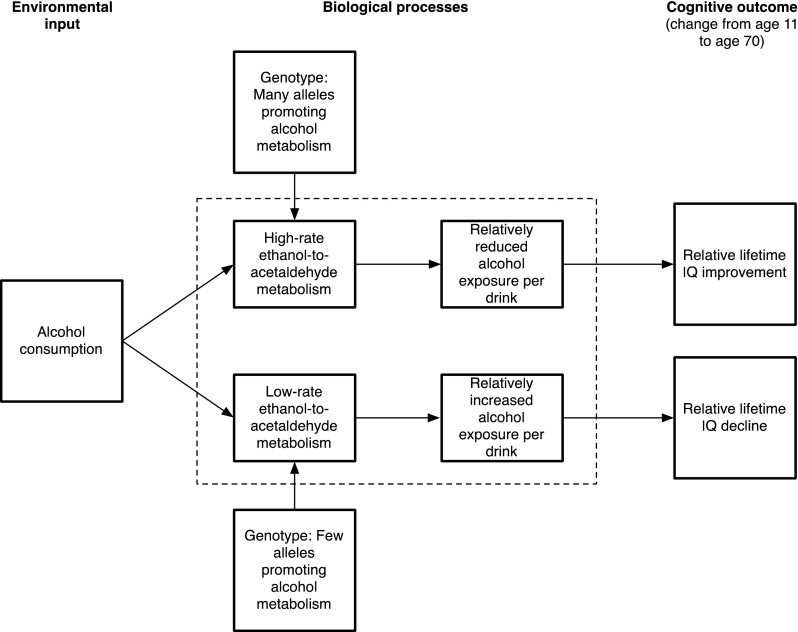
Theoretical model of alcohol’s effect on lifetime cognitive change. SNPs causing differences in alcohol metabolism interact with the alcohol consumption: Those with few rare alleles (and a corresponding higher rate of alcohol metabolism) have lower biological exposure to damaging effects of alcohol and thus a relative IQ improvement from alcohol consumption. The opposite effect occurs in those with many rare alleles, resulting in relative IQ decline. Variables inside the dashed box were not directly measured in the present study

Our theoretical model is illustrated in Fig. 1. We hypothesized that individuals with high SNP set scores (more rare alleles) would have lower alcohol metabolic capacity and would thus receive greater exposure to potentially damaging effects of alcohol. These individuals should have negative effects of alcohol consumption on the outcome variable and thus experience relatively more cognitive decline between ages 11 and 70. Conversely, individuals with low SNP set scores (fewer rare alleles) may metabolize the damaging factors in alcoholic drinks more efficiently and thus receive more of the theorized neuroprotective benefits that may also be present in such drinks (Collins et al. 2009). Specifically, we predicted an interaction between genotype and alcohol consumption, whereby low SNP scores lead to relatively beneficial effects on cognition with higher consumption, and high SNP scores lead to greater cognitive decline.

## Material and methods

### Participants

Participants were members of the Lothian Birth Cohort 1936 (LBC1936; Deary, Gow, Pattie, and Starr 2012), a sample of community-dwelling White European individuals, all of whom gave written consent for their involvement in the study. Ethics approval for the study was obtained from the Multi-Centre Research Ethics Committee for Scotland (MREC/01/0/56) and the Lothian Research Ethics Committee (LREC/2003/2/29).

Most of the LBC1936 members were tested for cognitive ability at a mean age of 10.94 years (SD = 0.28) during the Scottish Mental Survey of 1947. One thousand ninety-one individuals (543 females) were followed up between 2004 and 2007 (Deary et al. 2012), where they repeated the same cognitive test at a mean age of 69.53 years (SD = 0.83). Retaining only participants with mini-mental state examination (Folstein, Folstein, and McHugh 1975) scores above 24 (a commonly used cutoff for possible dementia; in the present study, we focused on nonpathological cognitive ageing) left 1,079 participants for analysis. Of these, 777 contributed all necessary variables for our analysis, including alcohol consumption, early and later cognitive abilities, covariates (parental and attained occupation-based SES, education level, and smoking status), and genotype. Analyses retaining participants with MMSE scores below 24 instead of including MMSE score as a covariate left the effects below largely unchanged.

### Phenotyping

Cognitive ability was measured twice, at ages ∼11 and ∼70 years, using the Moray House Test (MHT) No. 12 (Scottish Council for Research in Education 1958). Data were available from age ∼11 for 1,028 of the 1,079 participants who sat the later-life test. The test has 75 items and a total score of 76; the items consist of the following: following directions (14 items), same-opposites (11), word classification (10), analogies (8), practical knowledge (6), reasoning (5), proverbs (4), arithmetic (4), spatial abilities (4), mixed sentences (3), cypher decoding (2), other (4); see Deary, Whalley, Lemmon, Crawford, & Starr (2000) for more details. Scores on this test correlate strongly with IQ-type tests such as the Stanford-Binet test in childhood (*r* ∼ 0.8), and Raven’s Standard Progressive Matrices in old age (*r* ∼ 0.7) (Deary, Whiteman, Starr, Whalley, and Fox 2004). For the analyses below, MHT scores were—as is customary for IQ-type tests—standardized to a mean of 100 and a SD of 15. In addition, MHT scores were adjusted for age (in days) at the time of testing in childhood and old age.

Alcohol consumption was measured using a Food Frequency Questionnaire (FFQ; Masson et al. 2003), completed at the same session as later-life cognitive testing. Participants indicated how many standard measures of each type of alcohol they had consumed over the past 2–3 months. The questionnaire used is described in detail by Corley et al. (2011). Consumption was rated on a scale of one to nine: rarely or never, 1–3 per month, 1 per week, 2–3 per week, 4–6 per week, 1 per day, 2–3 per day, 4–6 per day, and >7 per day. This was done for each of nine classes of beverage: low-alcohol lager or beer; dark beer; light beer; white wine; red wine; sherry, port, etc.; spirits or liqueurs; ‘alcopops’; and cider. Alcohol consumption (in grams) per day was calculated from scores on each of these items by summing the grams of alcohol consumed for each of the nine beverage classes. Incomplete questionnaires, and one outlying score almost six SDs above the mean, were removed (including the outlying score made no substantive difference to the results reported below).

The alcohol intake variable was positively skewed (skewness = 2.05; many individuals with low consumption and fewer with very high consumption). Therefore, log-normalized total alcohol consumption was used in all analyses. Specifically, we used log (grams of alcohol/day) + 1, because some individuals reported no consumption.

Measures of four potential confounding variables were taken at interview at age ∼70. SES of origin was the social class of the job held by the participant’s father when they were born in 1936, on a scale from I (professional) to V (unskilled). The classification was obtained from the Office of Population Censuses and Surveys (1951). The participant’s achieved SES was the social class of the person’s principal and most prestigious job in adult life. This was on the same scale as SES of origin except that classification III was split into nonmanual (IIIN, recoded as 3.0) and manual (IIIM, recoded as 3.5; Office of Population Censuses and Surveys 1980). For female cohort members who worked, their husband’s job classification was used if it was of higher status than their own. Educational duration was the number of years of formal, full-time education experienced by the participant. Smoking status was recorded as one of three categories: “never smoked”, “ex-smoker”, and “current smoker”.

### Genotyping

The SNP set score was the number of minor alleles present for the four SNPs reported by Lewis et al. (2012): rs2866151, rs975833, rs284779, and rs4147536. Scores on the four-SNP set ranged from a possible minimum of zero rare alleles to a maximum of eight, theoretically corresponding to highest through to lowest ability to metabolize alcohol. Because of the imputation of three SNPs (see below), the score need not take integer values. DNA samples were genotyped at the Wellcome Trust Clinical Research Facility using the Illumina Human610-Quad v1.0 chip (Illumina, Inc., San Diego, CA). Five hundred forty-two thousand fifty SNPs passed quality control (see Houlihan et al. 2010). All SNPs included in the analyses had a call rate of ≥0.98, a minor allele frequency of ≥0.01, and a Hardy-Weinberg equilibrium test with *p* ≥ 0.001. Since the chip did not directly measure all genetic variants, imputation of ∼2.5 m common SNPs was conducted using MaCH v1.0.16 software (Li, Willer, Ding, Scheet, and Abecasis 2010) which uses HapMap phase II CEU full-genotype data (NCBI build 36 (UCSC hg18)) as the basis for an algorithm that imputes missing SNP data from theorized ancestral relationships. As Li et al. (2010) show, the MaCH imputation process is highly reliable and accurate. Three of the SNPs in the four-SNP set were imputed; rs4147536 was directly genotyped. Descriptive details about the four SNPs are provided in Table 1.

**Table 1:** Gene, alleles and minor-allele frequency (MAF), and imputation *R* ^2^ for each SNP

Gene	SNP rs-number	Alleles	MAF	Imputation *R* ^2^
*ADH1A*	rs2866151	A/T	0.452	0.987
	rs975833	G/C	0.216	0.999
*ADH1B*	rs4147536^a^	C/A	0.235	–
*ADH7*	rs284779	G/C	0.470	0.610

^a^Directly genotyped SNP; other SNPs imputed

### Statistical analysis

Gene × environment interaction between the four-SNP set and alcohol intake was tested for in a linear regression model with cognitive ability aged ∼70 as the dependent variable. The model included SNP set scores, log-alcohol intake, and the SNP set × alcohol consumption interaction term as predictors and six covariates: cognitive ability age ∼11, sex, years of education, smoking status, origin SES, and attained SES. After estimating the main model, we ran a number of checks of the robustness of the results: First, by including additional interaction terms as suggested by Keller (2013); second, by running the model without covariates; and third, by removing individuals with outlying cognitive change scores from the analysis.

## Results

**Fig. 2:**
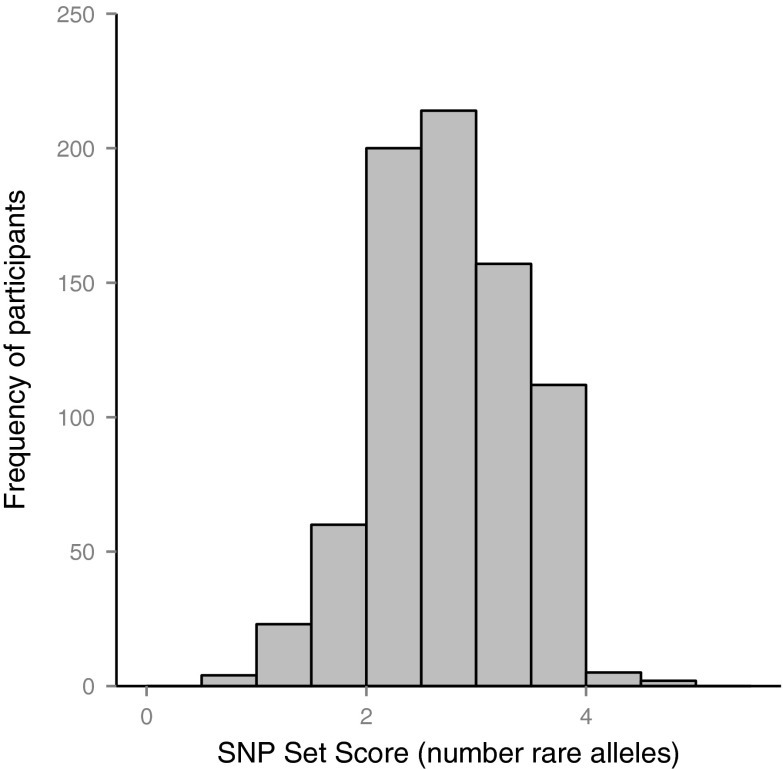
Histogram of four-SNP set scores (total number of rare/minor alleles from rs2866151, rs975833, rs4147536, and rs284779)

For the 777 individuals who contributed all variables required for the analysis, mean alcohol consumption per day as reported in the FFQ was 11.53 g (SD = 15.00), and the mean SNP score was 2.75 (SD = 0.69). The histogram in Fig. 2 shows that SNP scores were distributed approximately normally. The mean MHT score at age ∼11 was 50.22 (SD = 11.07) and at age ∼70 was 65.54 (SD = 7.62). Participants reported a mean of 10.79 years (SD = 1.12) of full-time education. Three hundred seventy participants (47.62 %) reported never having smoked, 333 (42.86 %) were ex-smokers, and 74 (9.52 %) were current smokers. The social class of participants’ fathers was distributed as follows: class I 6.56 % of the sample, class II 20.59 %, class III 55.98 %, class IV 9.14 %, and class V 7.72 %. For the social class of the participants’ own jobs, the distribution was as follows: class I 19.95 %, class II 38.35 %, class IIIN 15.96 %, class IIIM 22.78 %, class IV 2.57 %, and class V 0.39 %.

First, using linear regression models controlling for sex, we tested the assumptions of the MR design (Davey Smith 2010), namely that the genetic variable was unrelated to the potential confounders (origin and attained SES, education, and smoking status) and to the outcome variable (later-life cognition). SNP score was significantly positively associated with age ∼11 IQ (*b* = 1.65, *p* = 0.02) but was not significantly associated with SES of origin (*b* = 0.01, *p* = 0.85), attained SES (*b* = 0.05, *p* = 0.27), educational duration (*b* = −0.05, *p* = 0.89), smoking status (compared to “never smoked”, *b* = 0.07, *p* = 0.17 for ex-smokers and *b* = 0.05, *p* = 0.61 for current smokers) nor, importantly, with the main outcome variable, age ∼70 IQ (*b* = 1.02, *p* = 0.12). SNP score was not a significant predictor of change in cognitive ability between age ∼11 and age ∼70, calculated either by computing a residual score after controlling age ∼70 IQ for age ∼11 IQ (*b* = 0.02, *p* = 0.97), or by simply calculating the difference between two IQ scores (*b* = −0.63, *p* = 0.26). The genetic variable therefore met the assumptions of the MR design and analysis proceeded.

To test the hypothesis that alcohol intake is related to cognitive performance via ADH genotype, we ran the model described above in the “Statistical analysis” section. The results of the model are shown in Table 2. As predicted, a significant (albeit small) gene-environment interaction was found (*b* = −1.13, *p* = 0.007): alcohol consumption interacted with the genotype score to significantly predict age ∼70 cognitive ability.^1^ It should be noted that, in an otherwise identical model with no interaction term, neither alcohol consumption (*b* = −0.14, *p* = 0.62) nor SNP score (*b* = 0.11, *p* = 0.82) were significant predictors of age ∼70 cognitive ability.

**Table 2:** Linear regression model predicting age 70 cognitive ability (measured by the Moray House Test) from demographic variables, alcohol consumption, and four-SNP genotype score

Predictor	*b* (95 % CI)	SE	*t* value	*p* value
Sex	−2.65 (−4.04 to −1.27)	0.71	−3.75	<0.001
Age 11 cognitive ability (MHT)	0.55 (0.50 to 0.61)	0.03	20.85	<0.001
Origin SES	0.03 (−0.71 to 0.76)	0.38	0.08	0.93
Attained SES	−0.47 (−1.26 to 0.33)	0.41	−1.15	0.25
Years of education	1.37 (0.67 to 2.07)	0.36	3.83	<0.001
Smoking status: ex-smoker	0.34 (−1.05 to 1.74)	0.71	0.48	0.63
Smoking status: current smoker	−2.45 (−4.77 to −0.14)	1.18	−2.08	0.04
Alcohol consumption (log g per day)	2.93 (0.62 to 5.24)	1.17	2.50	0.01
Four-SNP score	2.40 (0.49 to 4.31)	0.97	2.47	0.01
Alcohol consumption × SNP score interaction	−1.13 (−1.94 to −0.31)	0.42	−2.72	0.007

Overall regression adjusted *R* ^*2*^ = 0.48, *F* (10,766) = 72.83, *p* < 0.001. For sex, negative coefficients reflect lower values for females. The alcohol consumption measure was log-normalized. SES variables were treated as continuous for the purposes of analysis. For smoking, “never smoked” was the reference category

*MHT* Moray House Test; *SES* socioeconomic status

**Fig. 3:**
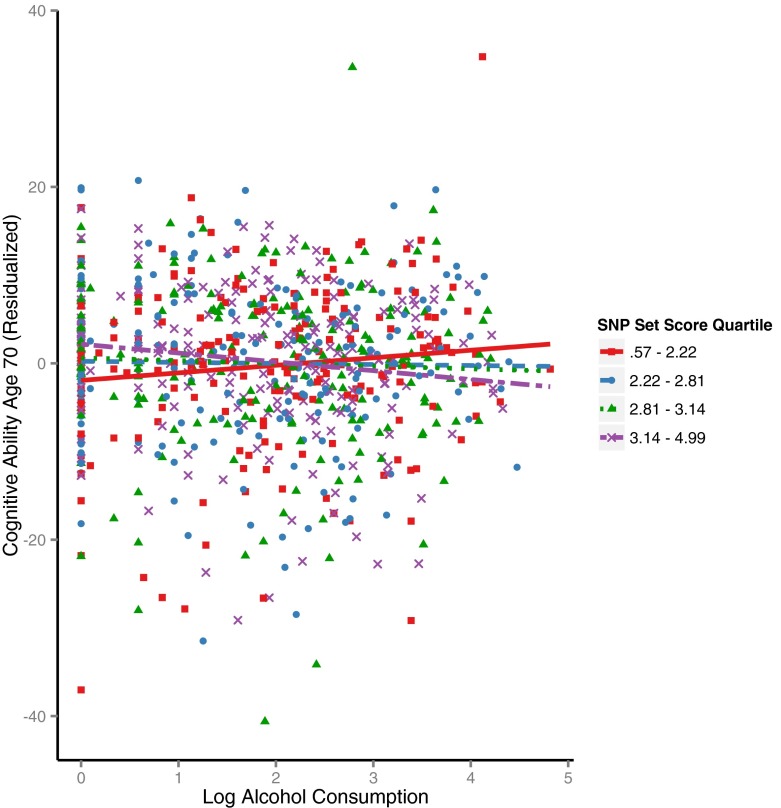
Lifetime cognitive change (age ∼70 cognitive ability adjusted for age ∼11 cognitive ability and confounders) against alcohol consumption, split by SNP set score quartile (quartiles are for illustration; note that analysis was performed on a continuous SNP score). Higher scores on the *y*-axis indicate better cognitive ability later in life. Higher SNP set scores indicate a greater number of rare alleles

Figure 3 shows the interaction. For illustrative purposes, it splits the SNP scores into quartiles, but all analyses were performed on the continuous SNP score variable. For those individuals with fewer rare alleles, the relationship between alcohol consumption and cognitive change was positive; this is shown in the upward-sloping line of best fit for the lowest SNP score quartile (solid line). Thus, as alcohol consumption rises, cognition (age 70 IQ adjusted for IQ at age 11) rises. Conversely, for those with more rare alleles, alcohol consumption was negatively associated with cognitive change, illustrated by the downward-sloping (dot-dash) line. As alcohol consumption rises, there is more relative lifetime cognitive decline. The lines for the two intermediate SNP score quartiles (dotted line and dashed line) are relatively flat, indicating no beneficial or detrimental effect of alcohol consumption on lifetime cognitive change for those individuals.

We ran a number of extra analyses to test the robustness of our results. First, Keller (2013) has argued that gene × environment analyses should include interactions not only between the genetic variants of interest and the environmental variable, but also between any covariates and the genetic variants and between covariates and the environmental variable. For this reason, we added twelve additional interaction terms—between each of the six covariates and the SNP set and between each covariate and log-alcohol consumption—to our model. The significant SNP score × log-alcohol consumption interaction remained (*b* = −1.17, *p* = 0.01). Second, we tested the robustness of our results to the removal of all covariates. A model including no covariates had an increased valid sample size of 803 and still showed a significant SNP score × log-alcohol consumption interaction (*b* = −0.72, *p* = 0.004); our result was thus not dependent on the inclusion of covariates.

Finally, Fig. 3 shows that two individuals with very large residualized cognitive ability changes appear above the majority of other participants, while several individuals lie below the majority. To assess the extent to which our effects were reliant on these outlying scores, we ran the analysis again, removing those with residualized cognitive ability increases or decreases of more than 30 IQ points. This analysis did not alter the main finding: a significant SNP score × log-alcohol consumption interaction remained (*b* = −1.08, *p* = 0.01 for a model including the interactions recommended by Keller 2013; *b* = −0.82, *p* = 0.037 for a model without these additional interactions). Thus, the gene × environment interaction effect was robust to the removal of individuals with outlying cognitive change scores.

## Discussion

This study examined the effect of alcohol consumption on cognitive ability in later life, testing the interaction of a four-SNP score indexing alcohol dehydrogenase activity with alcohol consumption. We controlled for cognitive ability prior to the start of consumption and thus studied lifetime cognitive change. We found a significant interaction between recent alcohol consumption and genetic differences in alcohol processing efficiency on IQ at age 70 adjusted for IQ at age 11. Inspection of this interaction indicated that, for subjects with high alcohol processing efficiency (lower SNP set score), greater consumption was associated with modest improvements in later-life intelligence. Conversely, for subjects with a lower ability to process alcohol (higher SNP set score), higher alcohol consumption was associated with loss of cognitive ability across the life course. Because the effects of alcohol operated via a SNP set that was itself unrelated to cognition or to social status variables, they are unlikely to be subject to the issues of reverse causation and confounding theorized to be present in previous analyses of this question (e.g. Corley et al. 2011).

The present result extends a previous finding, that maternal alcohol consumption is a risk factor for foetal cognitive development (Lewis et al. 2012), to the domain of the effects of alcohol consumption on an individual’s own lifetime cognitive change. It suggests that alcohol consumption may have either beneficial or deleterious effects on cognition, conditional on genotype. We found an interaction using a continuous measure of recent alcohol consumption; the effect did not, then, manifest only with moderate consumption. The finding was robust to controlling for outliers and to the presence or absence of covariates. One of the noteworthy outcomes of the study was to confirm the utility of the four-SNP set identified by Lewis et al. for future work. For instance, this set could be used to test the effect of alcohol consumption on other outcomes, such as cardiovascular disease risk (e.g. Hines et al. 2001) in a similar Mendelian randomization framework. In addition, our study adds to previous investigations of the effects of alcohol on later-life cognition that have looked at effects of alcohol across full samples (e.g. Corley et al. 2011) by identifying particular individual differences within those samples that may moderate alcohol’s effects.

The interactive effect of the four SNPs with alcohol found here was small; in this context, it should be recalled that the four SNPs in the set necessarily only explain a small portion of variance in alcohol metabolism activity (Birley et al. 2009). Because of this, it is possible that the total effect of the full range of alcohol exposure on cognitive outcomes is greater than the effect found here. Given the attenuation of the alcohol-cognition relation after partial control for reverse causation and confounding issues in previous studies, however, it is likely that the overall effect of (nonpathological) alcohol consumption on cognitive outcomes is modest. The true effect size can be assessed in future studies using SNP sets based on a deeper understanding of the genetics of alcohol metabolism.

Alcohol processing takes place in a range of bodily systems, including the digestive system (mediated by *ADH7*; Zgombic-Knight, Foglio, and Duester 1995), as well as in hepatic processing, which removes alcohol from the bloodstream. Future work should identify the precise biochemical mechanism by which the four SNPs identified here mediate the effects of alcohol on the brain. Other individual differences may also be relevant to the relationship discovered here. The present study controlled for sex in all analyses, and it would be inadvisable for reasons of statistical power, given the small effect discovered, to reanalyze each sex separately in this dataset. However, future studies in larger samples should test for differential effects by sex, and also by the range of other factors known to influence the relationship between alcohol and health, such as alcohol type and drinking pattern (e.g. Grønbæk 2009). Finally, given that acetaldehyde, the product of the breakdown of alcohol, has been linked to negative health outcomes such as increased cancer incidence (e.g. Brennan et al. 2004; Seitz and Stickel 2010), future research should take into account the potential deleterious effects of efficient alcohol processing as well as the possible cognitive benefits found here.

### Strengths and limitations

We analyzed data from a sample of individuals homogenous for age and culture, reducing any potential confounding influences of these factors. In addition, and importantly, cognition on the same ability test was measured both before and after most of each individual’s lifetime alcohol consumption. However, the present study also had limitations. First, the alcohol measure used in this study is a self-report of the amount of alcohol consumed per day over the previous 2–3 months. It cannot, therefore, give a full indication of lifetime alcohol consumption. We would expect that many of the individuals reporting no recent alcohol consumption on the FFQ at age 70 would have consumed alcohol throughout their life, even if in moderate amounts (this is especially likely since alcohol consumption tends to decline in older age, e.g. Brennan, Schutte, Moos, and Moos 2011); indeed, it is for this reason that we included the full sample, regardless of alcohol consumption, in our analyses. Future studies should, if possible, use an alcohol consumption measure obtained multiple times across the life course, as in a recent study by Sabia et al. (2014), who had available in their sample three measurements of alcohol taken in the decade preceding cognitive testing, although they did not perform a genetic analysis. If such measures are not practicable, alcohol use history measures, such as the Lifetime Drinking History questionnaire (Friesema et al. 2004) should be considered.

Second, our hypothesis (shown in Fig. 1), based on previous research (e.g. Dickson et al. 2006), suggested that the SNPs in the set we used indexed alcohol metabolism and thus the exposure to alcohol experienced by each participant. We did not, however, have available in our sample a measure of active exposure to alcohol; for instance, our study participants did not undergo an ‘alcohol challenge’, as used by Birley et al. (2009), where alcohol was consumed and blood and breath measures of alcohol concentration were taken. Our hypothesis should be tested in samples where such a measure is available.

## Conclusion

We showed that the effects of recent alcohol consumption vary from a reduction to an enhancement in later-life cognitive ability, contingent on an individual’s genetically influenced capacity to metabolize alcohol. These results validate a previously reported SNP set (Lewis et al. 2012), inform theory regarding the mechanisms by which alcohol affects cognition and are relevant to understanding the potential public health risks and benefits of alcohol consumption. Future research should attempt to replicate these findings using more detailed measures of alcohol consumption taken across the life course.

## Footnotes

1 At the request of a reviewer, we tested this interaction after removing the 108 individuals who reported zero alcohol consumption. This reduced the effect size of the gene × environment interaction to nonsignificance (*b* = −0.81, *p* = 0.11). However, individuals with no consumption are still part of our hypothesized interaction: those with more efficient genetic processing ability would be predicted to have better cognition if they consumed more alcohol. In addition, our alcohol measure did not record whether these participants were lifetime teetotal; they may have been alcohol drinkers earlier in life, even if at low levels (see “Strengths and limitations” below). The inclusion of these participants in the model is justified, since it avoids both restriction of the range of alcohol consumption and loss of statistical power by reducing the overall sample size.
